# Evidence for validity and reliability of a french version of the FAAM

**DOI:** 10.1186/1471-2474-12-40

**Published:** 2011-02-08

**Authors:** Stéphane Borloz, Xavier Crevoisier, Olivier Deriaz, Pierluigi Ballabeni, RobRoy L Martin, François Luthi

**Affiliations:** 1 Département de l'appareil locomoteur, Clinique Romande de réadaptation SUVA Care, Sion, Switzerland; 2 Département de l'appareil locomoteur, Centre Hospitalier Universitaire Vaudois and University of Lausanne, Lausanne, Switzerland; 3 Institut de recherche en réadaptation and Service de recherche médicale, Clinique Romande de réadaptation SUVA Care, Sion, Switzerland; 4 Institute of Social and Preventive Medicine (IUMSP), Centre Hospitalier Universitaire Vaudois and University of Lausanne, Lausanne, Switzerland; 5 Department of Physical Therapy, Duquesne University, Pittsburgh, PA, USA; 6 Center for Sports Medicine-University of Pittsburgh Medical Center Pittsburgh, PA, USA

## Abstract

**Background:**

The Foot and Ankle Ability Measure (FAAM) is a self reported questionnaire for patients with foot and ankle disorders available in English, German, and Persian. This study plans to translate the FAAM from English to French (FAAM-F) and assess the validity and reliability of this new version.

**Methods:**

The FAAM-F Activities of Daily Living (ADL) and sports subscales were completed by 105 French-speaking patients (average age 50.5 years) presenting various chronic foot and ankle disorders. Convergent and divergent validity was assessed by Pearson's correlation coefficients between the FAAM-F subscales and the SF-36 scales: Physical Functioning (PF), Physical Component Summary (PCS), Mental Health (MH) and Mental Component Summary (MCS). Internal consistency was calculated by Cronbach's Alpha (CA). To assess test re-test reliability, 22 patients filled out the questionnaire a second time to estimate minimal detectable changes (MDC) and intraclass correlation coefficients (ICC).

**Results:**

Correlations for FAAM-F ADL subscale were 0.85 with PF, 0.81 with PCS, 0.26 with MH, 0.37 with MCS. Correlations for FAAM-F Sports subscale were 0.72 with PF, 0.72 with PCS, 0.21 with MH, 0.29 with MCS. CA estimates were 0.97 for both subscales. Respectively for the ADL and Sports subscales, ICC were 0.97 and 0.94, errors for a single measure were 8 and 10 points at 95% confidence and the MDC values at 95% confidence were 7 and 18 points.

**Conclusion:**

The FAAM-F is valid and reliable for the self-assessment of physical function in French-speaking patients with a wide range of chronic foot and ankle disorders.

## Background

Evaluation of patients with musculoskeletal disorders can rely not only on clinical examination and radiological imaging, but also on scores from self-reported outcome instruments. The information acquired from self-reported outcome instruments is useful only if there is evidence to support interpretation of the obtained scores[1,2]. Currently there is a lack of evidence to support the use of an instrument in the French language for individuals with musculoskeletal foot and ankle disorders.

In the foot and ankle literature there are many instruments that have been implemented in outcome related research with little or no evidence to support their use[3,4]. Over 49 instruments have been identified[3] with 14 having some evidence to support their use[4]. Over these 14 instruments, the 4 clinimetric qualities (content validity, construct validity, reliability, and responsiveness) were only reached by the Foot and Ankle Ability Measure, the Foot Function index, the Foot Health Status Questionnaire, the Lower Extremity Function Scale, and the Sports Ankle Rating System quality of life measure[4]. Instruments that offer specific information for score interpretation may be more useful. This information can include score error associated with single measure, change in score that represents a minimal detectable change (MDC), and a change in score that represents minimal clinical important difference (MCID). The MDC quantifies the change in score value over time that is beyond measurement error. The MCID is a cut-off value over which changes discriminates between patients that have clinically improved from those that have not improved[4,5]. Even in instruments that have evidence to support their use, difficulties arise when they are applied to individuals who are not fluent in the language the instrument has been developed in. While there are many instruments available there is not a universally accepted instrument in the French language for individuals with musculoskeletal foot and ankle disorders.

The Foot and Ankle Ability Measure (FAAM), originally published in English[6], has evidence for reliability, responsiveness, and validity as measure of physical function. This evidence was provided in individuals with a wide range of musculoskeletal disorders of the lower leg, ankle, and foot and therefore has broad application. To evaluate activities that require a higher level of ability the FAAM also contains a Sports subscale[7]. Specific information for score interpretation, including MDC and MCID values are provided for the FAAM. Evidence to support its use of is also available for athletes with chronic ankle instability[8] and patients with Diabetes Mellitus[7]. Moreover, a cross cultural adaptation and validation has been completed to create German[9] and Persian versions of the FAAM[10].

Although there is increasing evidence to support the use of the FAAM, it has not been adapted and validated for French speaking individuals. In our clinical and research practice, most patients are French speaking, and not able to appropriately respond to items written in English. The aim of this study is to translate the FAAM into French, perform a cross-cultural adaptation, and provided evidence for validity and reliability for this French version of the FAAM (FAAM-F).

## Methods

### Foot and Ankle Ability Measure

The FAAM, as originally described, is comprised of separately scored 21-item Activity of Daily Living (ADL) and 8-item Sports subscales. The response to each item on the ADL subscale is scored from 4 to 0, with 4 being "no difficulty" and 0 being "unable to do". Items without a response or marked as not applicable are not counted. The score on each of the items are added together to get the item score total. The total number of items with a response is multiplied by 4 to get the highest potential score. If all 21 items are answered, the highest potential score is 84. If one item is unanswered the highest score was 80, if two were unanswered the total highest score was 76, etc. The total item score is divided by the highest potential score and then multiplied by 100 to produce the FAAM score that ranges from 0 to 100. The Sports subscale is scored in a similar manner the highest potential score is 32. As with the ADL subscale, the item score total is divided by the highest potential score and multiplied by 100. A higher score represents a higher level of physical function for both the ADL and Sports subscales[6].

### Cross-cultural adaptation

The cross-cultural adaptation of the FAAM was performed according to the guidelines of the American Academy of Orthopedic Surgeons (AAOS) Outcomes Committee and as recommended in the literature[11,12]. The following five steps were documented with a written report: 1) Forward translation from English to French by two translators native to French and fluent in English (T1 and T2). One of the translators was informed about the aims of the study, and the other received only limited information (so-called naïve translator). Moreover, the translators were not healthcare professionals. 2) Versions T1 and T2 were compared. A unique translated version (T12) was created by resolving any discrepancies under supervision of a methodologist who was not involved in the translation process. 3) Back translation of the T12 version from French into English by two translators native to English, and fluent in French (BT1 and BT2). These two translators were naïve to the study and not directly linked with the medical domain. 4) Consensus meeting with all the involved parties (translators, methodologist) and foot and ankle specialist physicians in order to resolve any discrepancies and doubts met during the translation, and to establish the pre-final French version of the FAAM. 5) Pre-testing of the French version of the FAAM in 40 consecutive patients with foot complaints, for the accuracy of wording and the ease of understanding. Patients were asked to mention any encountered difficulties during a phone interview. The last step included submitting the final version of the French FAAM-F and all reports and forms to a committee keeping track of the translated version and to the developer of the original instrument in order to verify that the recommended stages were followed.

### Patients

Consecutive ambulatory patients receiving treatment between June and August 2009 for an ankle or foot complaints were recruited for the study. These subjects were given a copy of the FAAM-F with a self-addressed return envelope, a written information about the study and a consent form. There were 139 patients seen either at the Clinique Romande de Réadaptation, Sion, Switzerland or the Foot & Ankle Department of the Centre Hospitalier Universitaire Vaudois (CHUV), Lausanne, Switzerland. These clinics are, respectively, a rehabilitation centre and a specialized orthopaedic department for foot and ankle disorders. Patients who had more than one missing item response on the FAAM-F were excluded from the study. No reminder was sent to those who did not return the questionnaires and the collection of the questionnaires has been stopped one month after having given the last one (n = 139). Out of the 139 patients 105 (76%) sent the questionnaires back. These 105 patients presented varied pathologies such as degeneration (e.g. ankle or midfoot osteoarthritis, tibialis posterior degeneration, acquired flatfoot), trauma (e.g. tibial pilon or calcaneal fractures, midfoot injuries, ankle sprains), congenital malformations (e.g. hallux valgus, coalition), inflammations (e.g. rheumatoid arthritis and other rheumatic disorders), complex regional pain syndrome and tumors. Demographic information for these 105 subjects are presented in Table 1. Symptom duration ranged from several months to several years. The study was approved by the ethical committees of Valais and Vaud, the federal states in which the two hospitals are located. All patients signed a written informed consent.

**Table 1:** Demographic Information

Age	50.5 years (range 18-82 SD 14.6 yrs)
**Gender**	
Female	64 (61%)
Male	41 (39%)
**Foot and Ankle Ability Measure Score**	
Activities of Daily Living subscale	74 (range 8-100 SD 22.1)
Sports subscale	44 (range 0-100 SD 31.0)
**Symptom Location**	
Hindfoot	55 (52%)
Forefoot	35 (33%)
Midfoot	6 (6%)
Global Foot	6 (6%).
Not Specified	3(3%)
**Diagnosis**	
Degenerative Disorders	48 (46%)
Traumatic or Post-traumatic	42 (39%)
Congenital	6 (6%)
Inflammatory/Complex Regional Pain Syndrome	3 (3%)
Tumor	1 (1%)
Not Specified	5 (5%)

### Evidence for Convergent and Divergent Validity

All patients completed the French version of the FAAM and the Medical Outcomes Short Form (SF-36)[13,14]. Convergent evidence was examined by assessing the correlation between the FAAM-F and SF-36 physical function (PF) subscale and physical component summary (PCS) scores using Pearson correlation coefficients. Divergent evidence was examined by assessing the correlation between the FAAM-F and SF-36 mental health (MH) subscale and the mental health component summary (MCS) scores. Testing for significant differences in the correlation coefficients between the FAAM-F and concurrent measures of physical function and mental health was done as previously described for our two-sided hypothesis[15]. The a priori type I error rate was set at 0.005 to account for the multiple comparisons.

### Evidence for Internal Structure and Consistency

Cronbach's coefficient alpha was calculated to assess internal consistency. The standard error of measure (SEM) of the FAAM scores was calculated as $\text{SEM}=\sigma \sqrt{\text{1-r}}$ where *σ *was the standard deviation of the scores and r was the coefficient alpha. A 95% CI was then calculated to determine the error associated with a score at a single point in time.

### Evidence for Test Re-Test Reliability

Twenty-two randomized patients (13 women and nine men) presenting chronic disorders, to whom the physician did not expected to have a significant improvement in the next two days (no intervention, therapy or drugs) were asked to fill out two FAAM-F at a two-day interval. Test re-test reliability was assessed using an ICC (2,1) with the initial and follow up FAAM-F scores. The SEM was calculated using the ICC reliability coefficient. The SEM was multiplied by $\sqrt{2}$ and a 95% CI was calculated to determine a value for minimal detectable change (MDC)[16].

Both subscales of the FAAM (ADL and Sports) were analyzed separately for validation criteria as described above. All calculations were performed using the software package Stata 11.0 for Windows, (StataCorp LP, 4905 Lakeway Drive, College Station, TX 77845, USA).

## Results

The response proportion was 76% (105 responders). The average FAAM-F score was 74 (range 8-100 SD 22.1) and 44 (range 0-100 SD 31.0) for the ADL and Sports subscales, respectively (Table 1). A total of 7 (6.7%) and 5 (4.8%) patients scored 100 (best possible score) on the ADL and Sports subscales, respectively. No patient scored 0 (worst possible score) for the ADL subscale and 6 patients (5.7%) scored 0 for the Sports subscale. Twenty-four patients (22.9%) had scores above 95 for the ADL subscale and 7 (6.7%) for the Sports subscale. Percentage of patients having scores below 5 for ADL and Sports subscales were 0% and 11.4% (12 patients), respectively.

### Cross cultural adaptation

The translation of the different items of the FAAM was carried out without any relevant difficulties. The back-translation of the T12 version in English was very similar to the original version. Only some typically American expressions were different as our back-translators were native from Great-Britain. During the phone interview with 40 patients for the pre-testing of the final French version, it appeared that the item "walking initially", translated first as "commencer à marcher", was understood in different ways by the patients. Therefore, this item was adapted into "faire les premiers pas (le matin au réveil/après une position assise prolongée)" in order to precise the question. Upon re-assessment with patients it was noted that this change improved item interpretation. No further difficulties in understanding the items were discovered during the pre-testing phase.

### Evidence for Convergent and Divergent Validity

For the convergent validity, we found correlation coefficients of 0.85 between the ADL and Physical Functioning, and 0.81 between ADL and PCS. The Sports subscale had correlations of 0.72 with both Physical Function and PCS. The assessment of divergent validity resulted in an ADL-Mental Health correlation of 0.26 and an ADL-MCS correlation of 0.37. The Sports subscale had a correlation of 0.21 with Mental Health and of 0.29 with MCS (Table 2). The calculated t-values assessing for difference in the correlation coefficients between the ADL and Sports subscales to measures of physical and mental functioning were significant with P < 0.0005.

**Table 2:** Correlation coefficients between the FAAM-F (ADL and Sports subscales) and the SF-36 (physical and mental scales).

	ADL subscale FAAM-F	Sport subscale FAAM-F	ADL subscale FAAM original	Sport subscale FAAM original	ADL subscale FAAM diabetes	Sport subscale FAAM diabetes
Physical Functioning	0.85 (n = 104)	0.72 (n = 102)	0.84 (n = 151)	0.78 (n = 130)	0.61 (n = 83)	0.63 (n = 83)

Physical Component Summary	0.81 n = 100)	0.72 (n = 98)	0.84 (n = 151)	0.80 (n = 130)	0.71 (n = 83)	0.72 (n = 83)

Mental Health	0.26 (n = 102)	0.21 (n = 100)	0.18 (n = 151)	0.11 (n = 130)	0.32 (n = 83)	0.22 (n = 83)

Mental Component Summary	0.37 (n = 100)	0.29 (n = 98)	0.05 (n = 151)	-0.02 (n = 130)	0.29 (n = 83)	0.17 (n = 83)

The two middle columns (FAAM original) present the values found by Martin et al. with the original English version, 2005[6]. The two right columns (FAMM diabetes) show the values found by Martin et al. 2009, using the original English version on patients suffering diabetes mellitus[7].

### Evidence of Internal Structure

The assessment of internal consistency found coefficient alpha to be 0.97 for both the ADL and Sports subscales. The score error associated with a single measure at 95% confidence was +/- 8 and +/- 10 points for the ADL and Sports subscale, respectively

### Evidence for Score Stability

The assessment of test re-test reliability over a two day period found ICC values of 0.97 and 0.94 for the ADL and Sports subscales, respectively. The MDC at 95% confidence was +/- 7 and +/- 18 points for the ADL and Sports subscales, respectively

## Discussion

The aims of this study were met as original English version of the FAAM was successfully translated and adapted into French to create the FAAM-F ADL and Sports subscales. Evidence for reliability and validity were offered to support the use of the FAAM-F as a self-report outcome instrument for individuals with a wide range of chronic foot and ankle disorders. Specifically, evidence for convergent validity, divergent validity, internal structure, and score stability were provided for the FAAM-F. Information for score interpretation with error associated with a single measure and MDC was also provided for the FAAM-F.

There is a lack of well designed, validated, questionnaire in French for assessing foot and ankle disorders [3]. Moreover, there is increasing evidence that self-questionnaires are useful for clinicians as well as for investigators in order to facilitate the patients' self evaluation. The FAAM is region specific measure, capable of being sensitive to changes in physical function. It is easy for patient to complete and uncomplicated for clinicians score, making it easy to add to the evaluation process. A systematic review concluded the FAAM and its predecessor were the most appropriate evaluative instruments to quantify functional disabilities in individuals with chronic ankle instability[17]. The FAAM has undergone advanced psychometric testing including the use of item response theory (IRT)[18]. The evidence to support the use of the FAAM as an outcome instrument continues to grow not only for the English version but for German and Persian versions as well. Therefore, it was felt the FAAM would be the most appropriate instrument to translate and adapt for French speaking individuals. Following the guidelines of the AAOS, the French adaptation of this questionnaire was performed without encountering any difficulties as only one of the 29 items had to be slightly modified for easier understanding (item related to "walking initially"). Moreover, the high response rate and the good feed-back during the phone interview demonstrated that the FAAM-F was well accepted.

The relationship between the FAAM-F and concurrent measures of physical function were significantly different than the associations between the FAAM-F and concurrent measures of mental health. This provides evidence that the FAAM-F is a measure of physical function as opposed to mental function. The coefficients of correlation between FAAM-F subscales and the SF-36 physical scales were high (0.85 and 0.72.). Moreover, they are very similar to the values found for the original version[6] (0.84 and 0.78; Table 2). The correlations between FAAM subscales and the SF-36 mental scales for divergent validity were low, as hypothesized and were similar to those for the original version with a good overlap of their 95% confidence intervals. The possible exception was noted for the ADL to MSC, 0.37 and 0.05 respectively for the French and in English versions (Table 2). This could be explained by some cultural difference regarding pain or type of foot disorders between the two studies. Our patients were recruited in tertiary centers and suffered for chronic disorders (months up to years), which can obviously affect mental function and self-perception. This explanation is also supported by the comparison of our results with the ones described in the publication with patients presenting with chronic foot complaints related to diabetes mellitus[7]. However, the correlation between ADL to MSC was low in our study, supporting our hypotheses for divergent validity.

The calculated MDC, for FAAM-F ADL and Sports subscales, differ from the values reported for the English version of the FAAM (ADL MDC 5,7 and 12,3; Sports MDC 7 and 18 for French and English versions, respectively). These discrepencies could be explained either by differences in patients population or by the time frame over which the MDC was calculated. The original study used a 4-week time period while the current study used a 2-day time period.

According to the literature, the CA value for a good internal consistency should be over 0.80 (over 0.90 for clinical applications)[19]. Despite the adaptation of one of the 21 items during cross-cultural-adaptation the internal consistency of the FAAM-F remained excellent and widely above the limit of acceptance with a CA value of 0.97 for ADL subscale. The Sports subscale as well showed an excellent internal consistency (0.97). These two values were almost identical to the values provided for the English version (0.98). Values noted for the error associated with a single measure, 8 and 10 points for the ADL and Sports subscales were also very similar to the English version, 7 and 10 points, respectively. The test re-test reliability of the FAAM-F was found to be excellent. The ICC values of this study were superior to those reported for the original version. This finding might be expected as the time interval in the original study was over a 4 week period compared to 2 days in the current study. The findings of this study help to confirm that the French translation is close to the original version[6].

When looking at the range of obtained scores, there were FAAM-F ADL and Sports scores with extreme high and low values. This could have potential problem with floor and ceiling effects if more than 15-20% of patients have scores in these values[2,5]. Twenty-four patients (22.9%) had scores above 95 for the ADL subscale and 7 (6.7%) for the Sports subscale. Therefore, a possible ceiling effect for the ADL subscale could be potentially problematic if used without the Sports subscale in individuals functioning at a high level. However, the ADL and Sports subscale were developed to be used together and complement one another. Therefore ceiling effects with the ADL subscale would be offset by the use of the Sports subscale which would collect information for those functioning at a high level of ability.

The limitations of this study must be recognized. Only patients with chronic foot disorders have been included in the present study. Patients with acute conditions may present differently. Furthermore, the responsiveness of the FAAM-F needs to be assessed with values for MCID offered to assist with score interpretation. It should be remembered that evidence to support the use of an instrument is an ongoing process with information needed under various study conditions. Furthermore, the enrolled patients were not randomly drawn from a population with chronic food and ankle disorders but were a convenience sample of available hospital patients.

## Conclusion

The results of this research offer evidence of reliability and validity for the FAAM-F for self-assessment of physical function in French-speaking patients with a wide range of chronic foot and ankle disorders.

## Competing interests

The authors declare that they have no competing interests.

## Authors' contributions

BS, CX, DO, LF and BP have designed the study. BS, CX and LF have gathered the data. BS, DO, BP, MRL and LF have analyzed the data. BS and MRL have written the initial draft. BS, CX, DO, BP, MRL and LF have ensured the accuracy of the data and analysis.

All authors read and approved the final manuscript.

## Pre-publication history

The pre-publication history for this paper can be accessed here:

http://www.biomedcentral.com/1471-2474/12/40/prepub
